# Hyperbolically Symmetric Versions of Lemaitre–Tolman–Bondi Spacetimes

**DOI:** 10.3390/e23091219

**Published:** 2021-09-16

**Authors:** Luis Herrera, Alicia Di Prisco, Justo Ospino

**Affiliations:** 1 Instituto Universitario de Física Fundamental y Matemáticas, Universidad de Salamanca, 37007 Salamanca, Spain; 2 Escuela de Física, Facultad de Ciencias, Universidad Central de Venezuela, Caracas 1050, Venezuela; alicia.diprisco@ciens.ucv.ve; 3 Departamento de Matemáticas Aplicada and Instituto Universitario de Física Fundamental y Matemáticas, Universidad de Salamanca, 37007 Salamanca, Spain; j.ospino@usal.es

**Keywords:** LTB spacetimes, hyperbolic symmetry, general relativity, dissipative systems, 04.40.-b, 04.20.-q, 04.40.Dg, 04.40.Nr

## Abstract

We study fluid distributions endowed with hyperbolic symmetry, which share many common features with Lemaitre–Tolman–Bondi (LTB) solutions (e.g., they are geodesic, shearing, and nonconformally flat, and the energy density is inhomogeneous). As such, they may be considered as hyperbolic symmetric versions of LTB, with spherical symmetry replaced by hyperbolic symmetry. We start by considering pure dust models, and afterwards, we extend our analysis to dissipative models with anisotropic pressure. In the former case, the complexity factor is necessarily nonvanishing, whereas in the latter cases, models with a vanishing complexity factor are found. The remarkable fact is that all solutions satisfying the vanishing complexity factor condition are necessarily nondissipative and satisfy the stiff equation of state.

## 1. Introduction

In a recent paper [1], we presented a general study on the dynamics of hyperbolic symmetric fluids (DHSFs). Our main motivation behind such an endeavor (but not the only one) was to describe the dynamic regime preceding the final equilibrium state of static hyperbolic symmetric fluids described in [2], which in its turn could be used to model the source of the hyperbolic symmetric black hole described in [3,4] where the region interior to the horizon is described by the line element:

(1) $$\begin{array}{ccc} ds^{2} & = & -\left(\frac{2M}{R}-1\right)dt^{2}+\frac{dR^{2}}{\left(\frac{2M}{R}-1\right)}+R^{2}d\Omega^{2},\\ d\Omega^{2} & = & d\theta^{2}+\sinh^{2}\theta d\phi^{2}. \end{array}$$

The rationale behind such a proposal stems from the well-known fact that any transformation that maintains the static form of the Schwarzschild metric (in the whole spacetime) is unable to remove the coordinate singularity in the line element [5]. In other words, the regular extension of the Schwarzschild metric to the whole spacetime (including the region inner to the horizon) may be achieved, but at the price of admitting a nonstatic spacetime inside the horizon [6,7].

Since any dynamic regime should eventually end in an equilibrium final state, it would be desirable to have a static solution over the whole spacetime.

Thus, the model proposed in [3] describes the space time as consisting of two four-dimensional manifolds, one described by the usual Schwarzschild metric on the exterior side of the horizon and a second one in the interior of it, described by (1).

The metric (1) is a static solution admitting the four Killing vectors:

(2) $$K_{\left(0\right)}=\partial_{t},$$

and:

(3) $$\begin{array}{c} K_{\left(2\right)}=-\cos\phi \partial_{\theta }+\coth\theta \sin\phi \partial_{\phi },\\ K_{\left(1\right)}=\partial_{\phi },\;K_{\left(3\right)}=\sin\phi \partial_{\theta }+\coth\theta \cos\phi \partial_{\phi }. \end{array}$$

The above Killing vectors (3) define the hyperbolic symmetry. Solutions to the Einstein equations endowed with this type of symmetry have been the subject of research in different contexts (see [8,9,10,11,12,13,14,15,16] and the references therein).

Besides the general properties of DHSFs analyzed in [1], some exact solutions were found. Particular attention is owed to two nondissipative solutions, which could be regarded as the hyperbolic symmetric versions of the Friedman–Robertson–Walker spacetime, since they are shear-free, nondissipative, and conformally flat. All solutions presented in [1] satisfy the condition of the vanishing complexity factor and evolve in the quasi-homologous regime. This last condition, in the nondissipative case, implies that the fluid is shear-free, thereby excluding the possibility to obtain a hyperbolic symmetric version of the LTB spacetimes under the above-mentioned conditions.

Due to the huge relevance of LTB spacetimes, we devote this work to study in some detail its possible hyperbolic symmetric versions. To do that, we must abandon the condition of quasi-homologous evolution.

It is worth recalling that LTB dust models [17,18,19] are among the most appealing solutions to the Einstein equations. They describe the spherically symmetric distribution of inhomogeneous nondissipative dust (see [20,21] for a detailed description of these spacetimes). Although LTB spacetimes are usually associated with an inhomogeneous dust source, it is known that the most general source compatible with LTB spacetimes is an anisotropic fluid [20,22].

LTB spacetimes have been invoked as cosmological models (see [23,24,25] and the references therein), in the study of gravitational collapse, when dealing with the problem of cosmic censorship [26,27,28,29,30,31], and in quantum gravity [32,33].

The apparent accelerated expansion of the universe, as inferred from some observations of type Ia supernovae, has renewed the interest in LTB spacetimes. Indeed, even though there is general consensus to invoke dark energy as a source of antigravity for understanding the cosmic acceleration, a growing number of researchers are now considering that inhomogeneities may account for the observed cosmic acceleration, without resorting to dark energy (see [34,35,36,37,38,39] and the references therein).

In this work, we present several models that could be considered as hyperbolic symmetric versions of LTB spacetimes, in the sense that they are geodesic, shearing, and nonconformally flat and the energy density is inhomogeneous, but with the spherical symmetry replaced by the hyperbolic symmetry.

We consider both nondissipative and dissipative models. The general approach used for reaching our goal was already outlined in [1]; however, for the sake of completeness, we present the basic steps in the following sections. The last section is devoted to the discussion of the obtained results.

## 2. Fluid Distribution, Kinematical Variables, and Basic Equations

We consider hyperbolic symmetric distributions of a geodesic fluid, which may be bounded (or not) from outside by a surface $\Sigma^{e}$. As we already know (see [1] for a detailed discussion on this point), hyperbolic symmetric fluids cannot fill the central region, and therefore, such a region should be described either by an empty vacuole or by a fluid distribution not endowed with hyperbolic symmetry. Thus, our fluid is also bounded from inside by a surface $\Sigma^{i}$.

Choosing comoving coordinates, the general metric can be written as:

(4) $$ds^{2}=-dt^{2}+B^{2}dr^{2}+R^{2}\left(d\theta^{2}+\sinh^{2}\theta d\phi^{2}\right),$$

where *B* and *R* are assumed positive and due to the symmetry defined by (3) are functions of *t* and *r*. We number the coordinates $x^{0}=t$, $x^{1}=r$, $x^{2}=\theta$, and $x^{3}=\phi$.

The general energy momentum tensor $T_{\alpha \beta }$ of the fluid distribution may be written as:

(5) $$\begin{array}{ccc} T_{\alpha \beta } & = & \left(\mu +P_{⊥}\right)V_{\alpha }V_{\beta }+P_{⊥}g_{\alpha \beta }+\left(P_{r}-P_{⊥}\right)\chi_{\alpha }\chi_{\beta }\\ & + & q_{\alpha }V_{\beta }+V_{\alpha }q_{\beta }, \end{array}$$

where μ is the energy density, $P_{r}$ the radial pressure, $P_{⊥}$ the tangential pressure, and $q^{\alpha }$ the heat flux; these physical variables, due to the symmetry defined by (3), are functions of *t* and *r*. Furthermore, $V^{\alpha }$ and $\chi^{\alpha }$ denote the four-velocity of the fluid and a unit four-vector along the radial direction, respectively; they satisfy:

(6) $$\begin{array}{c} V^{\alpha }V_{\alpha }=-1,\;\;V^{\alpha }q_{\alpha }=0,\;\;\chi^{\alpha }\chi_{\alpha }=1,\;\;\chi^{\alpha }V_{\alpha }=0. \end{array}$$

Since we consider comoving observers, we have:

(7) $$\begin{array}{c} V^{\alpha }=\delta_{0}^{\alpha },\;\;q^{\alpha }=qB^{-1}\delta_{1}^{\alpha },\;\;\chi^{\alpha }=B^{-1}\delta_{1}^{\alpha }. \end{array}$$

### 2.1. Einstein Equations

For (4) and (5), the Einstein equations:

(8) $$G_{\alpha \beta }=8\pi T_{\alpha \beta },$$

read:

(9) $$\begin{array}{c} 8\pi \mu =\left(2\frac{\dot{B}}{B}+\frac{\dot{R}}{R}\right)\frac{\dot{R}}{R}-{\left(\frac{1}{B}\right)}^{2}\left[2\frac{R^{\prime \prime }}{R}+{\left(\frac{R^{\prime }}{R}\right)}^{2}-2\frac{B^{\prime }}{B}\frac{R^{\prime }}{R}+{\left(\frac{B}{R}\right)}^{2}\right], \end{array}$$

(10) $$4\pi q=\frac{1}{B}\left(\frac{{\dot{R}}^{\prime }}{R}-\frac{\dot{B}}{B}\frac{R^{\prime }}{R}\right),$$

(11) $$\begin{array}{c} 8\pi P_{r}=-\left[2\frac{\ddot{R}}{R}+{\left(\frac{\dot{R}}{R}\right)}^{2}\right]+{\left(\frac{R^{\prime }}{BR}\right)}^{2}+{\left(\frac{1}{R}\right)}^{2}, \end{array}$$

(12) $$\begin{array}{c} 8\pi P_{⊥}=-\left(\frac{\ddot{B}}{B}+\frac{\ddot{R}}{R}+\frac{\dot{B}}{B}\frac{\dot{R}}{R}\right)+{\left(\frac{1}{B}\right)}^{2}\left(\frac{R^{\prime \prime }}{R}-\frac{B^{\prime }}{B}\frac{R^{\prime }}{R}\right), \end{array}$$

where dots and primes denote derivatives with respect to *t* and *r*, respectively. It is worth stressing the difference between these equations and those corresponding to the spherically symmetric LTB case.

### 2.2. Kinematical Variables and the Mass Function

The expansion Θ is given by:

(13) $$\Theta ={V^{\alpha }}_{;\alpha }=\left(\frac{\dot{B}}{B}+2\frac{\dot{R}}{R}\right),$$

and for the shear, we have (remember that the four-acceleration and the vorticity vanish):

(14) $$\sigma_{\alpha \beta }=V_{\left(\alpha ;\beta \right)}-\frac{1}{3}\Theta h_{\alpha \beta },$$

where $h_{\alpha \beta }=g_{\alpha \beta }+V_{\alpha }V_{\beta }$.

The nonvanishing components of (14) are:

(15) $$\sigma_{11}=\frac{2}{3}B^{2}\sigma ,\;\;\sigma_{22}=\frac{\sigma_{33}}{\sinh^{2}\theta }=-\frac{1}{3}R^{2}\sigma ,$$

with:

(16) $$\sigma^{\alpha \beta }\sigma_{\alpha \beta }=\frac{2}{3}\sigma^{2},$$

being:

(17) $$\sigma =\left(\frac{\dot{B}}{B}-\frac{\dot{R}}{R}\right),$$

$\sigma_{\alpha \beta }$ may also be written as:

(18) $$\sigma_{\alpha \beta }=\sigma \left(\chi_{\alpha }\chi_{\beta }-\frac{1}{3}h_{\alpha \beta }\right).$$

Next, the mass function m(t,r) introduced by Misner and Sharp [40] (see also [41]) is given by:

(19) $$m=-\frac{R}{2}R_{232}^{3}=\frac{R}{2}\left[-{\dot{R}}^{2}+{\left(\frac{R^{\prime }}{B}\right)}^{2}+1\right],$$

where the components $R_{232}^{3}$ of the Riemann tensor are calculated with (4).

Defining as usual the “areal” velocity *U* of the fluid as the variation of *R* with respect to proper time, i.e.,

(20) $$U=\dot{R},$$

then since U<1, it follows at once from (19) that *m* is a positive defined quantity.

With the above, we can express (19) as:

(21) $$E\equiv \frac{R^{\prime }}{B}={\left(U^{2}+\frac{2m}{R}-1\right)}^{1/2}.$$

From (19) and the field equations, we obtain:

(22) $$\begin{array}{c} \dot{m}=4\pi R^{2}\left(P_{r}U+qE\right), \end{array}$$

and:

(23) $$\begin{array}{c} m^{\prime }=-4\pi R^{\prime }R^{2}\left(\mu +q\frac{U}{E}\right). \end{array}$$

The integration of (23) produces:

(24) $$m=-\int_{0}^{r}4\pi R^{2}\left(\mu +q\frac{U}{E}\right)R^{\prime }dr,$$

whose partial integration yields:

(25) $$\frac{3m}{R^{3}}=-4\pi \mu +\frac{4\pi }{R^{3}}\int_{0}^{r}R^{3}\left(\mu^{\prime }-3q\frac{UB}{R}\right)dr.$$

Then, it follows from (24) that μ is necessarily negative, if we assume the condition $R^{\prime }>0$ to avoid shell crossing and remind that m>0.

Furthermore, it follows from (24) that whenever the energy density is regular, then $m∼r^{3}$ as *r* tends to zero. However, in this same limit, U∼0 and R∼r, implying because of (21) that the central region cannot be filled with our fluid distribution. Among the many possible scenarios, we shall assume here that the center is surrounded by a vacuum cavity. However, this is just one of the possible choices, which even if having implications on specific models, does not affect the general properties of the fluids endowed with hyperbolic symmetry.

### 2.3. The Exterior Spacetime and Junction Conditions

In the case of bounded configurations, we assume that outside $\Sigma^{e}$, we have the hyperbolic symmetric version of the Vaidya spacetime, described by:

(26) $$ds^{2}=-\left[\frac{2M(v)}{\rho }-1\right]dv^{2}-2d\rho dv+\rho^{2}\left(d\theta^{2}+\sinh^{2}\theta d\phi^{2}\right),$$

where M(v) denotes the total mass and *v* is the retarded time.

Now, from the continuity of the first differential form, it follows (see [42] for details),

(27) $$dt\overset{\Sigma^{e}}{=}dv\left(\frac{2M(v)}{\rho }-1\right),$$

(28) $$R\overset{\Sigma^{e}}{=}\rho \left(v\right),$$

and:

(29) $${\left(\frac{dv}{dt}\right)}^{-2}\overset{\Sigma^{e}}{=}\left(\frac{2M(v)}{\rho }-1+2\frac{d\rho }{dv}\right),$$

whereas the continuity of the second differential form produces:

(30) $$m\left(t,r\right)\overset{\Sigma^{e}}{=}M\left(v\right),$$

and:

(31) $$P_{r}\overset{\Sigma^{e}}{=}q,$$

where $\overset{\Sigma^{e}}{=}$ means that both sides of the equation are evaluated on $\Sigma^{e}$.

The corresponding junction conditions on $\Sigma^{i}$ are:

(32) $$m\left(t,r\right)\overset{\Sigma^{i}}{=}0,$$

and:

(33) $$P_{r}\overset{\Sigma^{i}}{=}0.$$

When either of the above conditions cannot be satisfied, we have to admit the presence of thin shells.

### 2.4. Weyl Tensor

The Weyl tensor is defined through the Riemann tensor $R_{\alpha \beta \mu }^{\rho }$, the Ricci tensor $R_{\alpha \beta }$, and the curvature scalar R as:

$$C_{\alpha \beta \mu }^{\rho }=R_{\alpha \beta \mu }^{\rho }-\frac{1}{2}R_{\beta }^{\rho }g_{\alpha \mu }+\frac{1}{2}R_{\alpha \beta }\delta_{\mu }^{\rho }-\frac{1}{2}R_{\alpha \mu }\delta_{\beta }^{\rho }$$

(34) $$+\frac{1}{2}R_{\mu }^{\rho }g_{\alpha \beta }+\frac{1}{6}R\left(\delta_{\beta }^{\rho }g_{\alpha \mu }-g_{\alpha \beta }\delta_{\mu }^{\rho }\right).$$

In our case, the magnetic part of the Weyl tensor vanishes, whereas its electric part, defined by:

(35) $$E_{\alpha \beta }=C_{\alpha \mu \beta \nu }V^{\mu }V^{\nu },$$

has the following nonvanishing components:

(36) $$\begin{array}{ccc} E_{11} & = & \frac{2}{3}B^{2}E,\\ E_{22} & = & -\frac{1}{3}R^{2}E,\\ E_{33} & = & E_{22}\sinh^{2}\theta , \end{array}$$

where:

(37) $$\begin{array}{ccc} & & E=\frac{1}{2}\left[\frac{\ddot{R}}{R}-\frac{\ddot{B}}{B}-\left(\frac{\dot{R}}{R}-\frac{\dot{B}}{B}\right)\frac{\dot{R}}{R}\right]\\ & + & \frac{1}{2B^{2}}\left[-\frac{R^{\prime \prime }}{R}+\left(\frac{B^{\prime }}{B}+\frac{R^{\prime }}{R}\right)\frac{R^{\prime }}{R}\right]+\frac{1}{2R^{2}}. \end{array}$$

Observe that we may also write $E_{\alpha \beta }$ as:

(38) $$E_{\alpha \beta }=E\left(\chi_{\alpha }\chi_{\beta }-\frac{1}{3}h_{\alpha \beta }\right).$$

Finally, using (9), (11) and (12) with (19) and (37), we obtain:

(39) $$\frac{3m}{R^{3}}=-4\pi \mu +4\pi \left(P_{r}-P_{⊥}\right)+E.$$

## 3. Structure Scalars and Complexity Factor

Some of the models exhibited below are obtained from the conditions imposed on a scalar function that appears in a natural way in the orthogonal splitting of the Riemann tensor (see [43] for details) and that is identified as the complexity factor.

Thus, let us introduce the tensor $Y_{\alpha \beta }$ (which is an element of that splitting [44,45,46,47]), defined by:

(40) $$Y_{\alpha \beta }=R_{\alpha \gamma \beta \delta }V^{\gamma }V^{\delta }.$$

Tensor $Y_{\alpha \beta }$ may be expressed in terms of two scalar functions $Y_{T},Y_{TF}$ (structure scalars) as:

(41) $$\begin{array}{c} Y_{\alpha \beta }=\frac{1}{3}Y_{T}h_{\alpha \beta }+Y_{TF}\left(\chi_{\alpha }\chi_{\beta }-\frac{1}{3}h_{\alpha \beta }\right), \end{array}$$

where:

(42) $$\begin{array}{c} Y_{T}=4\pi \left(\mu +3P_{r}-2\Pi \right),\;Y_{TF}=E-4\pi \Pi , \end{array}$$

with $\Pi =P_{r}-P_{⊥}$.

Combining (39) with (25) and (42) produces:

(43) $$Y_{TF}=-8\pi \Pi +\frac{4\pi }{R^{3}}\int_{0}^{r}R^{3}\left(\mu^{\prime }-3q\frac{UB}{R}\right)dr.$$

The complexity factor is a scalar function intended to measure the complexity of a given fluid distribution (see [48,49,50] for details). For static hyperbolic symmetric fluids (as well as for spherically symmetric ones), the complexity factor is identified with the scalar function $Y_{TF}$ defined, in the dynamic case, by Equations (42) and (43) (see [1]). The main reason behind such a proposal resides, on the one hand, in the basic assumption that one of the less complex systems corresponds to a homogeneous (in the energy density) fluid distribution with isotropic pressure. Thus, any variable measuring complexity should vanish for this specific case. On the other hand, the scalar function $Y_{TF}$ contains contributions from the energy density inhomogeneity and the local pressure anisotropy, combined in a very specific way, which (in the static case) vanishes for the homogeneous and locally isotropic fluid distribution. Furthermore, this scalar measures the departure of the value of the Tolman mass for the homogeneous and isotropic fluid, produced by the energy density inhomogeneity and the pressure anisotropy.

It is worth mentioning that the complexity factor so defined not only vanishes for the simple configuration mentioned above, but also may vanish when the terms appearing in its definition cancel each other. Thus, vanishing complexity may correspond to very different systems.

In the time-dependent case, we face two different problems: on the one hand, we have to generalize the concept of the complexity of the structure of the fluid distribution to time-dependent dissipative fluids, and on the other hand, we also have to evaluate the complexity of the mode of evolution. Following the strategy outlined in [49], the complexity factor for the dissipative case of the fluid distribution is assumed to be the function $Y_{TF}$, as in the static case, which now includes the dissipative variables. With respect to the complexity of the mode of evolution, let us recall that in the past, the homologous and quasi-homologous conditions have been used to characterize the simplest mode of evolution. However, we know that in the nondissipative case, the homologous and the quasi-homologous conditions imply the vanishing of the shear (see Equation (59) in [1]), and therefore, we shall not adopt such restrictions here.

## 4. Hyperbolically Symmetric Lemaitre–Tolman–Bondi Metric: The Nondissipative Dust Case

We start our search of hyperbolic symmetric versions of LTB (HSLTB) by considering the simplest case, i.e., we assume nondissipative geodesic dust. Extensions to the dissipative, anisotropic case are discussed in the next section, along the lines developed in [51].

Under the above-mentioned conditions, we find after integration of (10):

(44) $$B\left(t,r\right)=\frac{R^{\prime }}{{\left[k(r)-1\right]}^{1/2}},$$

where *k* is an arbitrary function of *r*.

Then, from (19) and (44), it follows:

(45) $${\dot{R}}^{2}=-\frac{2m}{R}+k\left(r\right).$$

Equation (45) implies $k\left(r\right)>\frac{2m}{R}$. Thus, unlike the spherically symmetric LTB spacetime, we now have only one case k(r)>0.

The solution to (45) may be written as:

(46) $$R=\frac{m}{k}\left(\cosh\eta +1\right),\;\frac{m}{k^{3/2}}\left(\sinh\eta +\eta \right)=t-t_{0}\left(r\right),$$

where $t_{0}\left(r\right)$ is an integration function of *r*.

Thus, for the line element, we have:

(47) $$ds^{2}=-dt^{2}+\frac{{\left(R^{\prime }\right)}^{2}}{k(r)-1}dr^{2}+R^{2}\left(d\theta^{2}+\sinh^{2}\theta d\phi^{2}\right).$$

In order to prescribe an explicit model, we have to provide the three functions k(r), m(r), and $t_{0}\left(r\right)$. However, since (47) is invariant under transformations of the form $r=r(\tilde{r})$, we only need two functions of *r*.

Assuming $m_{0}=\frac{m}{k}=constant$ and $t_{0}\left(r\right)=constant$, the expressions for Θ and σ read:

(48) $$\begin{array}{ccc} \Theta & = & \frac{\sqrt{k}}{m_{0}}\left(\frac{\sinh\eta }{\sinh\eta +\eta }+\frac{\cosh\eta +2\sinh\eta +1}{{\left(\cosh\eta +1\right)}^{2}}\right), \end{array}$$

(49) $$\begin{array}{ccc} \sigma & = & \frac{\sqrt{k}}{m_{0}}\left(\frac{\sinh\eta }{\sinh\eta +\eta }+\frac{\cosh\eta -\sinh\eta +1}{{\left(\cosh\eta +1\right)}^{2}}\right), \end{array}$$

from which it is clear that the expansion is always positive.

Since, as we have already mentioned, our fluid distribution cannot reach the central region, then we do not need to consider any regularity conditions there.

The only nontrivial conservation law in this case reads:

(50) $$\dot{\mu }+\mu \Theta =0,$$

or:

(51) $$\begin{array}{c} \dot{\mu }+\mu \left(\frac{\dot{B}}{B}+2\frac{\dot{R}}{R}\right)=0, \end{array}$$

producing:

(52) $$\mu =\frac{h(r)}{BR^{2}},$$

or, using (44):

(53) $$\mu =\frac{3h\left(r\right){\left[k(r)-1\right]}^{1/2}}{{\left(R^{3}\right)}^{\prime }},$$

where h(r) is a function of integration, which due to the fact that the energy density is negative, must be necessarily negative.

Scalar $Y_{TF}$ for (47) reads:

(54) $$Y_{TF}=\frac{\ddot{R}}{R}-\frac{{\ddot{R}}^{\prime }}{R^{\prime }}.$$

As is evident from (43), since we are considering nondissipative inhomogeneous dust, the complexity factor $Y_{TF}$ cannot vanish. However, this situation may change in the dissipative, anisotropic case, as we see in the models exhibited below. On the other hand, since in the nondissipative case, the quasi-homologous condition implies the vanishing of the shear (see Equation (59) in [1]), we have to abandon such a restriction for our models.

## 5. Dissipative Case

We now consider the possibility that the system radiates, and the pressure is nonvanishing and may be anisotropic. To do so, following the approach presented in [51], let us assume:

(55) $$B\left(t,r\right)=\frac{R^{\prime }}{\sqrt{K(t,r)-1}},$$

then integrating (10), we find:

(56) $$K\left(t,r\right)-1={\left[\int 4\pi qRdt+C(r)\right]}^{2},$$

since in the nondissipative case, (55) becomes (44), then $C\left(r\right)=\sqrt{k(r)-1})$.

Thus, the line element reads:

(57) $$ds^{2}=-dt^{2}+\frac{{\left(R^{\prime }\right)}^{2}dr^{2}}{{\left[\int 4\pi qRdt+C(r)\right]}^{2}}+R^{2}\left(d\theta^{2}+\sinh^{2}\theta d\phi^{2}\right).$$

Since we are considering dissipative systems, we need a transport equation. For simplicity, we adopt here the transport equation ensuing from the so-called “truncated” theory [52]; it reads:

(58) $$\tau h^{\alpha \beta }V^{\gamma }q_{\beta ;\gamma }+q^{\alpha }=-\kappa h^{\alpha \beta }T_{,\beta },$$

whose only nonvanishing independent component becomes:

(59) $$\tau \dot{q}+q=-\frac{\kappa }{B}T^{\prime },$$

where κ and τ denote the thermal conductivity and the relaxation time, respectively.

In order to obtain specific models, we need to impose additional conditions. A first family of models is obtained from conditions on the complexity factor, while a second family is obtained by a specific restriction on the function *B*, particularly suitable for describing situations where a cavity surrounding the central region appears.

### 5.1. Models Obtained upon Conditions on the Complexity Factor

In our case, the complexity factor $Y_{TF}$ may be written as:

(60) $$\begin{array}{c} Y_{TF}=\frac{\ddot{R}}{R}-\frac{{\ddot{R}}^{\prime }}{R^{\prime }}+\frac{\ddot{K}}{2(K-1)}+\frac{\dot{K}}{K-1}\left(\frac{{\dot{R}}^{\prime }}{R^{\prime }}-\frac{3}{4}\frac{\dot{K}}{K-1}\right). \end{array}$$

In order to obtain the models, we shall assume that the above structure scalar has the same form as in the nondissipative case, implying:

(61) $$\frac{\ddot{K}}{2(K-1)}+\frac{\dot{K}}{K-1}\left(\frac{{\dot{R}}^{\prime }}{R^{\prime }}-\frac{3}{4}\frac{\dot{K}}{K-1}\right)=0.$$

The integration of (61) produces:

(62) $$\frac{R^{\prime }\sqrt{\dot{K}}}{{\left(K-1\right)}^{\frac{3}{4}}}=C_{1}\left(r\right),$$

where $C_{1}$ is an integration function. Integrating the above equation, we obtain:

(63) $$K-1=\frac{4}{{\left[-C_{1}^{2}\int \frac{dt}{{\left(R^{\prime }\right)}^{2}}+C_{2}\left(r\right)\right]}^{2}},$$

where $C_{2}$ is another integration function.

Combining (56) and (63), it follows that:

(64) $$\int 4\pi qRdt+C\left(r\right)=\frac{2}{-C_{1}^{2}\int \frac{dt}{{\left(R^{\prime }\right)}^{2}}+C_{2}\left(r\right)},$$

or:

(65) $$C_{2}\left(r\right)=\frac{2}{\int 4\pi qRdt+C(r)}+C_{1}^{2}\int \frac{dt}{{\left(R^{\prime }\right)}^{2}}.$$

Using (62), it follows that $C_{2}\left(r\right)=0$; thus:

(66) $$\frac{2}{\int 4\pi qRdt+C(r)}+C_{1}^{2}\int \frac{dt}{{\left(R^{\prime }\right)}^{2}}=0,$$

implying that (64) may be written as:

(67) $$2\pi q=\frac{1}{R{\left(R^{\prime }C_{1}\left(r\right)\int \frac{dt}{{\left(R^{\prime }\right)}^{2}}\right)}^{2}}.$$

Let us first try to obtain models of dissipative dust satisfying (63).

Then, using (55) and (61), we obtain:

(68) $$\frac{\dot{B}}{B}=\frac{{\dot{R}}^{\prime }}{R^{\prime }}-\frac{\dot{K}}{{2(K-1)}^{’}}\;\frac{\ddot{B}}{B}=\frac{{\ddot{R}}^{\prime }}{R^{\prime }}.$$

Feeding back (68) in (11) and (12) produces:

(69) $$\begin{array}{ccc} K & = & 2\ddot{R}R+{\dot{R}}^{2}, \end{array}$$

(70) $$\begin{array}{ccc} K^{\prime } & = & 2R^{\prime }R\left[\frac{{\ddot{R}}^{\prime }}{R^{\prime }}+\frac{\ddot{R}}{R}+\frac{\dot{R}}{R}\left(\frac{{\dot{R}}^{\prime }}{R^{\prime }}-\frac{C_{1}^{2}\sqrt{K-1}}{2{\left(R^{\prime }\right)}^{2}}\right)\right]. \end{array}$$

Next, taking the *r* derivative of (69) and replacing it in (), we find:

(71) $$\frac{\dot{R}}{R}\frac{C_{1}^{2}\sqrt{K-1}}{2{\left(R^{\prime }\right)}^{2}}=0,$$

from which it follows at once that there are no radiating dust solutions in this case. Therefore, in the following subsection, we relax the dust condition, and we consider models of radiating anisotropic fluids.

#### $P_{⊥}=0$, $P_{r}\neq 0$

Let us consider models with vanishing tangential pressure.

Replacing (55) in (12), the following expression for $P_{⊥}$ is found:

(72) $$8\pi P_{⊥}=-\left[\frac{\ddot{B}}{B}+\frac{\ddot{R}}{R}+\frac{\dot{R}}{R}\left(\frac{{\dot{R}}^{\prime }}{R^{\prime }}-\frac{\dot{K}}{2(K-1)}\right)\right]+\frac{K^{\prime }}{2RR^{\prime }}.$$

In order to obtain a model, let us choose:

(73) $$\frac{\dot{R}}{R}\frac{{\dot{R}}^{\prime }}{R^{\prime }}=\frac{K^{\prime }}{2RR^{\prime }},$$

implying:

(74) $$K-1={\dot{R}}^{2}.$$

Then, from the condition $P_{⊥}=0$, we obtain from (72):

(75) $$\ddot{B}=0,\;\Rightarrow B=b_{1}\left(r\right)t+b_{2}\left(r\right),$$

where $b_{1}$ and $b_{2}$ are two arbitrary functions.

Using (55), we find for *R*:

(76) $$R^{\prime }-B\dot{R}=0,\;\Rightarrow \;R=\Phi \left[a_{1}\left(r\right)t+a_{2}\left(r\right)\right],$$

where Φ is an arbitrary function of its argument: and

(77) $$a_{1}\left(r\right)=e^{\int b_{1}\left(r\right)dr},\;a_{2}\left(r\right)=\int b_{2}\left(r\right)e^{\int b_{1}\left(r\right)dr}dr.$$

Then, the physical variables read:

(78) $$\begin{array}{ccc} 8\pi \mu & = & -\frac{1}{R^{2}}-\frac{\dot{R}}{R}\frac{\dot{K}}{\left(K-1\right)}, \end{array}$$

(79) $$\begin{array}{ccc} 4\pi q & = & \frac{\dot{K}}{2R\sqrt{K-1}}, \end{array}$$

(80) $$\begin{array}{ccc} 8\pi P_{r} & = & -\frac{\dot{R}}{R}\frac{\dot{K}}{K-1}+\frac{1}{R^{2}}. \end{array}$$

To further specify the model, let us choose $b_{1}\left(r\right)$ and $b_{2}\left(r\right)$ as:

(81) $$b_{1}\left(r\right)=\frac{\beta_{1}}{r+\beta_{2}},\;b_{2}\left(r\right)={\left(r+\beta_{2}\right)}^{\alpha },$$

implying:

(82) $$a_{1}\left(r\right)={\left(r+\beta_{2}\right)}^{\beta_{1}},\;a_{2}\left(r\right)=\frac{{\left(r+\beta_{2}\right)}^{\alpha +\beta_{1}+1}}{\alpha +\beta_{1}+1},$$

and:

(83) $$\begin{array}{ccc} B & = & \frac{\beta_{1}t}{r+\beta_{2}}+{\left(r+\beta_{2}\right)}^{\alpha }, \end{array}$$

(84) $$\begin{array}{ccc} R & = & {\left(a_{1}t+a_{2}\right)}^{n}, \end{array}$$

where $\beta_{1}$, $\beta_{2}$, α, and *n* are arbitrary constants.

Thus, the physical and kinematical variables for this model read:

(85) $$\begin{array}{ccc} 8\pi \mu & = & -\frac{1}{{\left(a_{1}t+a_{2}\right)}^{2n}}-\frac{2\left(n-1\right)na_{1}^{2}}{{\left(a_{1}t+a_{2}\right)}^{2}}, \end{array}$$

(86) $$\begin{array}{ccc} 4\pi q & = & \frac{\left(n-1\right)na_{1}^{2}}{{\left(a_{1}t+a_{2}\right)}^{2}}, \end{array}$$

(87) $$\begin{array}{ccc} 8\pi P_{r} & = & \frac{1}{{\left(a_{1}t+a_{2}\right)}^{2n}}-\frac{2\left(n-1\right)na_{1}^{2}}{{\left(a_{1}t+a_{2}\right)}^{2}}, \end{array}$$

(88) $$\begin{array}{ccc} m & = & \frac{{\left(a_{1}t+a_{2}\right)}^{n}}{2}, \end{array}$$

(89) $$\begin{array}{ccc} \Theta & = & \frac{b_{1}}{b_{1}t+b_{2}}+\frac{2na_{1}}{a_{1}t+a_{2}}, \end{array}$$

(90) $$\begin{array}{ccc} \sigma & = & \frac{b_{1}}{b_{1}t+b_{2}}-\frac{na_{1}}{a_{1}t+a_{2}}. \end{array}$$

A simple calculation of the complexity factor ($Y_{TF}$) for this model produces:

(91) $$Y_{TF}=\frac{\left(n-1\right)na_{1}^{2}}{{\left(a_{1}t+a_{2}\right)}^{2}}.$$

It is worth noticing that it has exactly the same expression as *q* as given by (). Therefore, any solution of this family satisfying the vanishing complexity factor is necessarily nondissipative. On the other hand, $Y_{TF}$ is zero if n=0 and/or $a_{1}=0$ and/or n=1. The first two conditions are ruled out at once from (82) and (85). Thus, the solution of this family with a vanishing complexity factor is characterized by n=1, which using (85) and (), produces:

(92) $$P_{r}=-\mu .$$

The above is the stiff equation of state originally considered by Zeldovich (see [48]).

### 5.2. Models with B=1

We next assume B=1 in order to obtain some additional analytical models. As discussed in [53], such a condition is particularly suitable for describing fluid distributions whose center is surrounded by an empty cavity, a scenario we expect for the kind of fluid distributions we are dealing with in this work.

The corresponding Einstein equations may be written as:

(93) $$\begin{array}{ccc} 8\pi \mu & = & -\frac{1}{R^{2}}-\frac{2R^{\prime \prime }}{R}-{\left(\frac{R^{\prime }}{R}\right)}^{2}+\frac{{\dot{R}}^{2}}{R^{2}}, \end{array}$$

(94) $$\begin{array}{ccc} 4\pi q & = & \frac{{\dot{R}}^{\prime }}{R}, \end{array}$$

(95) $$\begin{array}{ccc} 8\pi P_{r} & = & \frac{1}{R^{2}}+{\left(\frac{R^{\prime }}{R}\right)}^{2}-\left[{\left(\frac{\dot{R}}{R}\right)}^{2}+\frac{2\ddot{R}}{R}\right], \end{array}$$

(96) $$\begin{array}{ccc} 8\pi P_{⊥} & = & \frac{R^{\prime \prime }}{R}-\frac{\ddot{R}}{R}. \end{array}$$

#### 5.2.1. Nondissipative Case

Let us first consider the nondissipative case (q=0). In this case, it follows at once from (94) that *R* is a separable function, i.e., it takes the form:

(97) $$R=R_{1}\left(t\right)+R_{2}\left(r\right),$$

where $R_{1}$ and $R_{2}$ are arbitrary functions of their arguments.

Using (97) in (44), it follows at once that:

(98) $$R_{2}^{\prime }=\sqrt{k(r)-1}.$$

In order to exhibit an exact solution, let us further assume $P_{⊥}=0$. Using this condition in (96) produces:

(99) $$R_{1}\left(t\right)=at^{2}+b_{1}t+c_{1},\;R_{2}\left(r\right)=ar^{2}+b_{2}r+c_{2},$$

where $a,b_{1},c_{1},b_{2},c_{2}$ are arbitrary constants.

The physical and kinematical variables for this model are:

(100) $$8\pi \mu =\frac{1}{\alpha^{2}}\left(-1-4a\alpha -\beta^{2}+\gamma^{2}\right),$$

(101) $$8\pi P_{r}=\frac{1}{\alpha^{2}}\left(1-4a\alpha +\beta^{2}-\gamma^{2}\right),$$

(102) $$P_{r}+\mu =-\frac{a}{\pi \alpha },$$

(103) $$\Theta =\frac{2\gamma }{\alpha },$$

(104) $$\sigma =-\frac{\gamma }{\alpha },$$

(105) $$m=\frac{\alpha }{2}\left(\beta^{2}-\gamma^{2}+1\right),$$

where:

(106) $$\begin{array}{ccc} \alpha & \equiv & a\left(t^{2}+r^{2}\right)+b_{1}t+b_{2}r+c_{1}+c_{2};\;\beta \equiv 2ar+b_{2},\\ & & \gamma \equiv 2at+b_{1}. \end{array}$$

For this model, the expression for $Y_{TF}$ reads:

(107) $$Y_{TF}=\frac{2a}{\alpha }.$$

Therefore, the vanishing complexity factor implies a=0, which using (100) and (101) produces:

(108) $$P_{r}=-\mu .$$

Thus, the solution of this family with the vanishing complexity factor condition is also characterized by the stiff equation of state.

#### 5.2.2. Dissipative Case

Let us now consider the dissipative case (q≠0). If we impose the condition $P_{⊥}=0$, then we obtain the equation $\ddot{R}=R^{\prime \prime }$, whose general solution is of the form:

(109) $$R\left(t,r\right)=c_{1}\Psi \left(t+r\right)+c_{2}\Phi \left(t-r\right),$$

where $c_{1},c_{2}$ are arbitrary constants and Ψ,Φ arbitrary functions of their arguments.

As an example, let us choose:

(110) R(t,r)=csina(t−r),

where a,c are constants. Then, for the kinematical and physical variables, we obtain:

(111) $$8\pi \mu =2a^{2}-\frac{1}{c^{2}\sin^{2}\left[a\left(t-r\right)\right]},$$

(112) $$4\pi q=a^{2},$$

(113) $$8\pi P_{r}=2a^{2}+\frac{1}{c^{2}\sin^{2}\left[a\left(t-r\right)\right]},$$

(114) Θ=2acot[a(t−r)],

(115) σ=−acot[a(t−r)],

(116) $$m=\frac{c\sin[a(t-r)]}{2},$$

For this case, the temperature T(t,r), calculated from (59), reads:

(117) $$T\left(t,r\right)=-\frac{a^{2}r}{4\pi \kappa }+T_{0}\left(t\right),$$

whereas the expression for $Y_{TF}$ is:

(118) $$Y_{TF}=-a^{2},$$

implying because of (110) that no solution of this family has a vanishing complexity factor.

Finally, as an alternative model, we may assume:

(119) $$R=a{\left(t-r\right)}^{n},$$

where a,n are constants.

The ensuing physical and kinematical variables are in this case:

(120) $$8\pi \mu =-\frac{1}{a^{2}{\left(t-r\right)}^{2n}}-\frac{2n\left(n-1\right)}{{\left(t-r\right)}^{2}},$$

(121) $$4\pi q=-\frac{n\left(n-1\right)}{{\left(t-r\right)}^{2}},$$

(122) $$8\pi P_{r}=\frac{1}{a^{2}{\left(t-r\right)}^{2n}}-\frac{2n\left(n-1\right)}{{\left(t-r\right)}^{2}},$$

(123) $$\Theta =\frac{2n}{t-r},$$

(124) $$\sigma =-\frac{n}{t-r},$$

(125) $$m=\frac{a{\left(t-r\right)}^{n}}{2}.$$

Using (59), the expression for the temperature becomes:

(126) $$T\left(t,r\right)=\frac{n(n-1)}{4\pi \kappa (t-r)}-\frac{n(n-1)\tau }{4\pi \kappa {\left(t-r\right)}^{2}}+T_{0}\left(t\right).$$

The complexity factor for this family of solutions reads:

(127) $$Y_{TF}=\frac{n(n-1)}{{\left(t-r\right)}^{2}}.$$

The above scalar may vanish only if n=0 and/or n=1. The first possibility has to be ruled out from a simple inspection of (120), and therefore, the vanishing complexity factor conditions require n=1, implying because of (121) that the fluid is nondissipative and because of (120) and (122) that the fluid satisfies the stiff equation of state $P_{r}=-\mu$.

## 6. Discussion and Conclusions

We investigated in some detail all possible solutions of fluids endowed with the hyperbolic symmetry (3), characterized by nonvanishing shear, inhomogeneous energy–density, and vanishing four-acceleration (geodesics). So defined, these solutions are entitled to be considered as hyperbolic symmetric versions of LTB spacetimes.

The first class of solution corresponds to nondissipative dust configurations. Comparing with the spherically symmetric case, we observe that only one family of solutions (k(r)>0) exists, instead of the three families existing in this latter case (k(r)⪋0).

These solutions cannot satisfy the vanishing complexity factor, neither can they evolve in the quasi-homologous regime. On the other hand, the scalar expansion is positive as expected for pure dust submitted to a repulsive gravity.

Next, we analyzed the case of dissipative anisotropic fluids. To do this, we generalized the expression (44) by assuming (55). Different specific models were found from two different conditions. One class of solutions was obtained from a condition imposed on the complexity factor (61). It was shown that in this case, the pressure must be anisotropic. A solution of this type was found assuming further that $P_{⊥}=0$. The subclass of this solution satisfying the vanishing complexity factor is necessarily nondissipative and satisfies the stiff equation of state $P_{r}=-\mu$.

The other class of solutions was found under the condition B=1. For the nondissipative case, a family of solutions was found under the additional condition $P_{⊥}=0$. In this case as well, the vanishing complexity factor condition implies the stiff equation of state $P_{r}=-\mu$. In the dissipative case, two families of solutions were found from different assumptions on the specific form of *R*. Thus, assuming (110), we found a solution never satisfying the vanishing complexity factor condition, whereas assuming (119), such a condition can be satisfied, implying that the fluid is nondissipative and satisfies the stiff equation of state $P_{r}=-\mu$. It is worth noticing that the temperature for the first of the above solutions (117) does not contain terms depending on the relaxation time. In other words, the model behaves as if the fluid is always in the thermal stationary state, a result that becomes intelligible when we observe that the dissipative flux (112) is constant. Instead, for the second family of solutions, the temperature (126) clearly exhibits the effects of transient phenomena (i.e., those depending on τ).

Finally, we would like to conclude with a general comment: all the models exhibited above were found with the sole purpose of illustrating the richness of solutions endowed with hyperbolic symmetry and sharing the general physical and geometrical properties (excluding the isometry group) characterizing the LTB spacetimes. It is now up to cosmologists and astrophysicists to decide if any of the above models (or any other HSLTB not described in this manuscript) could be of any use in the study of specific scenarios, as for example cosmological models beyond the standard FRW solution [54,55]. 

## Data Availability

Not applicable.
